# The Microbial Detection Array for Detection of Emerging Viruses in Clinical Samples - A Useful Panmicrobial Diagnostic Tool

**DOI:** 10.1371/journal.pone.0100813

**Published:** 2014-06-25

**Authors:** Maiken W. Rosenstierne, Kevin S. McLoughlin, Majken Lindholm Olesen, Anna Papa, Shea N. Gardner, Olivier Engler, Sebastien Plumet, Ali Mirazimi, Manfred Weidmann, Matthias Niedrig, Anders Fomsgaard, Lena Erlandsson

**Affiliations:** 1 Department of Microbiological Diagnostics and Virology, Statens Serum Institut, Copenhagen, Denmark; 2 Global Security, Lawrence Livermore National Laboratory, Livermore, California, United States of America; 3 Department of Microbiology, Aristotle University of Thessaloniki, Thessaloniki, Greece; 4 Spiez Laboratory, Federal Office for Civil Protection, Spiez, Switzerland; 5 Virology department, French Army Forces Biomedical Institute (IRBA), Marseille, France; 6 Swedish Institute for Communicable Disease Control, Solna, Sweden; 7 National Veterinary Institute (SVA), Uppsala, Sweden; 8 Department of Clinical and Experimental Medicine, Linköping University, Linköping, Sweden; 9 Institute of Aquaculture, University of Stirling, Stirling, United Kingdom; 10 Centre for Biosafety, Robert Koch-Institute, Berlin, Germany; 11 Institute of Clinical Research, University of Southern Denmark, Odense, Denmark; University of California, San Francisco, United States of America

## Abstract

Emerging viruses are usually endemic to tropical and sub-tropical regions of the world, but increased global travel, climate change and changes in lifestyle are believed to contribute to the spread of these viruses into new regions. Many of these viruses cause similar disease symptoms as other emerging viruses or common infections, making these unexpected pathogens difficult to diagnose. Broad-spectrum pathogen detection microarrays containing probes for all sequenced viruses and bacteria can provide rapid identification of viruses, guiding decisions about treatment and appropriate case management. We report a modified Whole Transcriptome Amplification (WTA) method that increases unbiased amplification, particular of RNA viruses. Using this modified WTA method, we tested the specificity and sensitivity of the Lawrence Livermore Microbial Detection Array (LLMDA) against a wide range of emerging viruses present in both non-clinical and clinical samples using two different microarray data analysis methods.

## Introduction

Emerging viruses may be defined as viruses that are newly discovered or have the potential to increase in incidence or geographical range. Some important emerging viruses cause severe acute syndromes such as viral haemorrhagic fevers or encephalitides. They are endemic to tropical and sub-tropical regions. The majority are RNA viruses, from the *Arenaviridae, Bunyaviridae, Filoviridae, Flaviviridae* and *Togaviridae* virus families, but some are from DNA virus families such as *Poxviridae*. Their survival often depends on a vertebrate or arthropod host (non-human primates, bats, birds, rodents, ticks, sandflies or mosquitoes) [1]–[4]. They are usually restricted to geographical areas where the host species lives. Human cases occur through zoonosis, often resulting in life-threatening diseases with high mortality rates [3]. Knowledge of some of these viruses is limited, and originates exclusively from case reports and animal models. Some of them were first described during surveillance of veterinary diseases, e.g. Usutu virus, and only later became implicated in human clinical cases [5], [6].

Due to increased global travel, lifestyle changes and climate change, the risk of importing rare, exotic and emerging diseases to Europe has increased [3]. Some areas in Europe already maintain environmental conditions favourable to these pathogens, e.g. hantavirus [7], Crimean-Congo haemorrhagic fever virus (CCHFV) [8] and West Nile virus (WNV) [9]. Travellers visiting endemic areas are a potential source for spreading these diseases, which manifest as febrile illness coinciding with the peak of viral shedding and consequent risk of transmission. Disease symptoms may be nonspecific and similar to those of other common diseases, making them clinically difficult to recognize and diagnose [10]. There is a demand for rapid and accurate identification of the virus to initiate specific treatment, if available, as well as appropriate case management such as isolation and contact tracking [10], [11]. The use of real-time PCR has been critical for case management and epidemiological investigation, complementing serological diagnostic tools [12]. However, a PCR assay can only detect the presence of a specific virus, or a small group of viruses, and real-time PCR multiplexing is limited by overlapping fluorophore emission spectra and available detection channels in real-time PCR cyclers [13].

Several metagenomic approaches, such as microarrays [14]–[16], resequencing microarrays [17] and next generation sequencing [18], have been shown to be promising new tools for broad-spectrum diagnosis of common viral infections [19]–[21], arboviral diseases [22] and tropical febrile illnesses [23], [24]. These methods all have the ability to simultaneously test for the presence of thousands of viruses in a single assay and thereby remove the need for a specific clinical hypothesis regarding a suspected pathogen.

The Lawrence Livermore Microbial Detection Array (LLMDA) is a high density oligonucleotide microarray that contains probes for all sequenced viruses and bacteria [14]. It has been used to detect a wide range of viruses in both clinical samples [19], [25] and vaccine products [26]. In this study we report a modified Whole Transcriptome Amplification (WTA) protocol that increases the unbiased amplification of viruses, especially RNA viruses. Using this method we show that the version 2 of the LLMDA (LLMDAv2) is sensitive and specific to a wide range of emerging viruses and successfully identifies emerging virus present in clinical samples. In addition we compare the simpler SSI-developed data analysis method with the more sophisticated CLiMax software developed especially for LLMDA arrays.

## Materials and Methods

### Ethics Statement

Exemption for review by the ethical committee system and informed consent was given by the Committee on Biomedical Research Ethics-Capital region in accordance with Danish law on assay development projects.

### Data Availability Statement

All authors comply with the data availability policy.

### Virus from Non-clinical Samples

Within the European Network for Diagnostics of Imported Viral Diseases (ENIVD) we gathered a wide range of emerging viruses, as inactivated culture supernatants or as purified viral DNA or RNA (Table 1). Viruses were inactivated by heat and/or gamma-irradiation, or by suspension in an RNA-extraction reagent (TRIzol, Life Technologies; TriFast, Peqlab; AVL buffer, Qiagen) [27]. The majority of viruses were grown in Vero E6 cell-cultures (kidney epithelial cell line derived from African green gonkey) (ATCC CRL-1586), but poliovirus (PV) was grown in L20B cells (a murine recombinant cell line) [28]. We also used six control samples from the QCMD EQA programme for 2010 and 2013 (WNV10-01, WNV10-07, WNV13-01, WNV13-10, WNV13-11 and DENV13-01). The WNV13-01 sample contained West Nile virus (WNV) at a concentration of 1.0×10^7^ copies/ml and the DENV13-01 sample contained Dengue virus (DENV) type 1 at a concentration of 1.0×10^6^ copies/ml. The WNV10-01 and WNV13-10 samples contained a mixture of flaviviruses (DENV type 1, 2 and 4, and Japanese encephalitis virus (JEV)). The WNV10-07 and WNV13-11 samples contained a mixture of DENV type 3, tick-borne encephalitis virus (TBEV) and yellow fever virus (YFV), each at a concentration of 1.0×10^6^ copies/ml.

**Table 1:** Microarray detection range on WT-amplified samples.

Sample type	Sample	Conc^a^	Volume^b^	RT-reaction^c^	Before WTA^c^	After WTA^a^	ΔC_t_ *	Fold increase^+^	Microarray detection
SN	RVFV-6	3.3×10^6^	1	3.6×10^5^	1.8×10^5^	3.4×10^9^	20–19	34	na
	RVFV-5	3.3×10^5^		3.6×10^4^	1.8×10^4^	3.2×10^9^	22–19	154	na
	RVFV-4	3.3×10^4^		3.6×10^3^	1.8×10^3^	8.7×10^8^	26–21	474	+
	RVFV-3	3.3×10^3^		360	180	4.1×10^8^	29–22	2.7×10^3^	+
	RVFV-2	330		36	18	1.7×10^5^	34–34	25	ND
WNV13-01	WNV-7	1.2×10^7^	0.6	2.9×10^6^	1.4×10^5^	4.3×10^8^	20–14	1.7×10^3^	na
	WNV-6	1.2×10^6^		2.9×10^5^	1.4×10^4^	6.3×10^6^	24–22	122	+
	WNV-5	1.2×10^5^		2.9×10^4^	1.4×10^4^	1.8×10^9^	30–25	1.1×10^3^	+
	WNV-4	1.2×10^4^		2.9×10^3^	1.4×10^3^	6.7×10^10^	36–17	9.9×10^6^	+
	WNV-3	1.2×10^3^		290	140	1.6×10^6^	41–38	185	+^95^
	WNV-2	120		29	14	4.3×10^5^	42–41	34	ND
WNV13-10§	JEV-6§	1.0×10^6^	0.6	2.4×10^5^	1.2×10^5^	3.0×10^8^	20–17	320	+
	JEV-5§	1.0×10^5^		2.4×10^4^	1.2×10^4^	4.1×10^6^	23–25	10	+
	JEV-4§	1.0×10^4^		2.4×10^3^	1.2×10^3^	1.5×10^6^	28–27	52	+
	JEV-3§	1.0×10^3^		240	120	1.0×10^4^	33–36	4	ND
WNV13-11#	TBEV-6#	1.0×10^6^	0.6	2.4×10^5^	1.2×10^5^	3.6×10^6^	23–28	1	+
	TBEV-5#	1.0×10^5^		2.4×10^4^	1.2×10^4^	ND	28-ND	-	+
	TBEV-4#	1.0×10^4^		2.4×10^3^	1.2×10^3^	3.4×10^4^	33–37	1	+
	TBEV-3#	1.0×10^3^		240	120	2.1×10^4^	37–38	7	ND
	YFV-6	1.0×10^6^		2.4×10^5^	1.2×10^5^	7.5×10^5^	27–34	0,1	+
	YFV-5	1.0×10^5^		2.4×10^4^	1.2×10^4^	ND	31-ND	−	+
	YFV-4	1.0×10^4^		2.4×10^3^	1.2×10^3^	ND	36-ND	−	ND
	YFV-3	1.0×10^3^		240	120	ND	ND	−	ND
DENV13–01	DENV-6	1.0×10^6^	0.6	2.4×10^5^	1.2×10^5^	8.4×10^9^	28–19	1.3×10^4^	+
	DENV-5	1.0×10^5^		2.4×10^4^	1.2×10^4^	7.8×10^8^	31–23	8.4×10^3^	+
	DENV-4	1.0×10^4^		2.4×10^3^	1.2×10^3^	1.1×10^5^	35–39	2	+
	DENV-3	1.0×10^3^		240	120	3.1×10^5^	40–37	247	ND
Serum	HCV-6	1.2×10^6^	1	2.9×10^5^	1.4×10^5^	3.8×10^8^	26–14	1.3×10^5^	na
	HCV-5	1.2×10^5^		2.9×10^4^	1.4×10^4^	5.1×10^8^	29–13	1.3×10^6^	+
	HCV-4	1.2×10^4^		2.9×10^3^	1.4×10^3^	1.1×10^11^	33–15	6.4×10^6^	+
	HCV-3	1.2×10^3^		290	140	7.5×10^7^	36–26	3.0×10^4^	+
	HCV-2	120		29	14	4.4×10^5^	39–33	1.4×10^3^	ND

**NOTE**. Conc, Concentration; SN, supernatant; RVFV, Rift-Valley fever virus; WNV13-01, sample from QCMD EQA WNV panel 13-01; WNV, West Nile virus; JEV, Japanese encephalitis virus; TBEV, tick borne encephalitis virus; DENV13-01, sample from QCMD EQA DENV panel 13-01; DENV, Dengue virus; HCV, hepatitis C virus; ND, not detected; na, not analysed.

§ WNV13-10 contain additional viruses (DENV-1, DENV-2 and DENV-4).

# WNV13-11 contain addition viruses (YFV, DENV-3).

95 results obtained using a 95 percentile threshold.

a copies/ml (HCV; IU/ml).

b ml (volume of sample for purification).

c Number of copies into reaction.

*Difference in C_t_-value in real-time PCR before and after WT amplification.

+Fold increase after WT amplification, calculated from ΔC_t_ combined with dilution factors for each sample.

### Virus from Clinical Samples

We used clinical samples received for routine diagnostic analysis at Statens Serum Institut (SSI), Copenhagen, Denmark (Danish National reference laboratory (ISO 17025; 2005)), from the CCH Fever Project bio-bank at the Swedish Institute for Communicable Disease Control (Sweden), and from the Department of Microbiology, Aristotle University of Thessaloniki (Greece). The samples were (Table 2): i) One parapoxvirus-positive skin lesion (blister) sample from the hands of a shepherd; ii) One Chikungunya virus-positive serum sample from a traveller hospitalized for Dengue-like symptoms (high fever, joint pain, rash) after visiting Thailand; iii) Eight DENV-positive serum samples from travellers experiencing mosquito bites in the jungle of Thailand, iv) One CCHFV-positive serum sample (from the CCH Fever program); v) One sandfly fever Toscana virus-positive cerebrospinal fluid (CSF) sample from a traveller hospitalized with meningitis after visiting Toscana, Italy; vi) Six WNV-positive urine samples from patients hospitalized with West Nile fever (two of them with encephalitis). In addition, we used six hepatitis C virus (HCV)-positive serum samples and five HCV-positive plasma samples. One of the HCV-positive serum samples had a known viral concentration (1.2×10^6^ IU/ml) determined by standardisation against the WHO control. As negative controls we used virus-negative clinical samples (urine, CSF and serum).

**Table 2:** Microarray results on non-clinical samples using two different data analysis methods.

Group*	Genus	Virus	Sample	Detected virus SSI analysis	Detected virus CliMax analysis
dsDNA	Orthopoxvirus	Cowpox	pur. DNA	**Cowpox virus**, Variola virus, Monkeypox virus, Vaccinia virus, HERV	**Cowpox virus**, Variola minor virus∧, BEV, HERV
		Monkeypox	Pur. DNA	**Monkeypox virus,** Variola virus, Cowpox virus, Vaccinia virus, HERV	**Monkeypox virus**, Variola minor virus∧, BEV, HERV
(+) ssRNA	Alphavirus	EEEV	SN	**EEEV**, HERV	**EEEV**, BEV, HERV, SRV-1∧
	Flavivirus	Usutu	pur. RNA	**Usutu virus**, HERV, JEV	**Usutu virus,** BEV, HERV
		WNV	pur. RNA	**WNV**, HERV	**WNV,** BEV, HERV
		JEV, DENV-2, DENV-1, DENV-4	WNV10-01	**JEV, DENV-2, DENV-1, DENV-4**	**JEV, DENV-2, DENV-1, DENV-4,** DENV-3, BVDV-1∧, RV-A, PRV-C
		TBE, DENV-3, YF	WNV10-07	**TBEV, DENV-3, YFV**, DENV-1, DENV-2, OHFV, HERV	**TBEV, DENV-3, YFV,** DENV-2, SV5, RV-A, PRV-C
	Enterovirus	PV-1, PV-2	SN	**PV-1, PV-2**, PV-3	**PV-1, PV-2**, MuLV, SV40, MDEV, MMTV
(−) ssRNA	Arenavirus	Lassa	SN	**Lassa virus**	**Lassa virus**
	Hantavirus	DOBV	SN	**DOBV**	**DOBV**
		Hantaan	SN	**Hantaan virus**	**Hantaan virus,** MRV-3, MRV-1, MuLV
		Puumala	SN	**Puumala virus**, HERV	**Puumala virus,** BEV, HERV, BVDV-1∧
		Seoul	SN	**Seoul virus**, HERV	**Seoul virus**
		Sin Nombre	SN	**Sin Nombre**virus, HERV	**Sin Nombre virus,** BEV, HERV, SRV-1
	Nairovirus	CCHF	SN	**CCHFV**, HERV	**CCHFV,** HERV, BEV
	Phlebovirus	RVF	SN	**RVFV**, HERV	**RVFV,** CCHFV, SV5, BEV, HERV
		Naples	SN	**Naples virus**	**Naples virus,** BVDV-1
		Sicilian	SN	**Sicilian virus**	**Sicilian virus**
		Toscana	SN	**Toscana virus**, HERV	**Toscana virus,** BEV, HERV, SRV-1
	Ebolavirus	Ebola Zaire	SN	**Ebola Zaire virus**, HERV	**Ebola Zaire virus**, HERV, BEV, SRV-1
	Marburgvirus	Marburg	SN	**Marburg virus**, HERV	**Marburg virus,** HERV, BEV, RVFV∧

**NOTE**. EEEV, Eastern equine encephalitis virus; WNV, West Nile virus; CCHFV, Crimean-Congo haemorrhagic fever virus; RVFV, Rift-Valley fever virus; TBEV, Tick borne encephalitis virus; OHFV, Omsk hemoratic fever virus; YFV, yellow fever virus; PV, poliovirus; HERV, human endogenous retrovirus; JEV, Japanese encephalitis virus; DENV, Dengue virus; DOBV, Dobrava-Belgrade virus; RV-A, rotavirus A; PRV-C, porcine rotavirus C; BEV, baboon endogenous virus; SRV-1, simian retrovirus 1; MuLV, murine leukemia virus; SV40, simian virus 40; MDEV, *mus dunni* endogeneous virus; MMTV, mouse mammary tumour virus; MRV, mammalian orthoreovirus; BVDV, bovine viral diarrhea virus; SV5, simian virus 5; pur. DNA, purified DNA; SN, cell culture supernatant; pur. RNA, purified RNA; WNV10-01, sample from QCMD EQA WNV panel 10-01; WNV10-07, sample from QCMD EQA WNV panel 10-07 Bold represents correctly identified virus.

∧Viruses with fragmented alignment plots.

*Viruses are grouped based on nucleic acid content, according to the Baltimore Classification.

### Purification of Samples

As previously described [19] we centrifuged 230 µl of sample at 17,000 g for 10 min, filtered the supernatant through a 0.22 µm Spin-X spin filter (Costar) and treated it with DNase (Invitrogen or New England Biolabs) for 30 min-1½ h. The viral nucleic acid (NA) was extracted using the PureLink Viral RNA/DNA kit (Invitrogen), without the addition of carrier RNA. All samples were treated with this protocol with the exception of the QCMD panel samples, CSF, urine, and plasma samples, which were not DNase treated. Virus-positive supernatants suspended in RNA-extraction reagent were purified according to the manufacturer’s instructions (TRIzol, Life Technologies; TriFast, Peqlab; AVL buffer, Qiagen). The resulting RNA was further purified using the QIAamp RNA viral Mini kit (Qiagen). The extracted viral NA was eluted with 30–50 µl DNase/RNase-free water, and stored at −20°C or immediately used.

### Reverse Transcription

Reverse transcription (RT) on purified viral RNA was performed with three different methods: i) The P-N6/SSIII method, which uses the Superscript III Reverse Transcription kit (Invitrogen), combined with 5′-phosphorylated random hexamers (P-N_6_) (Eurofins MWG Operon). Briefly, 11–12 µl viral RNA was mixed with 1 µl 10 mM dNTP mix and 1 µl 250 ng/µl P-N_6_, incubated at 85°C for 5 min, and cooled on ice. Next, 4 µl 5x first strand buffer, 1 µl 0.1 M DTT, 1 µl RNaseOUT (40 U/µl) (optional) and 1 µl Superscript III RT enzyme (200 U/µl) was added, and the sample mixed and incubated at 25°C for 10 min, 42°C for 60 min and 95°C for 5 min. ii) The RT-reaction included in the WTA kit (Qiagen), which uses T-Script reverse transcriptase combined with random and oligo-dT primers. RT was performed according to the manufacturer’s instructions. iii) The VILO method, which uses a cDNA Synthesis kit (Invitrogen) containing Superscript III reverse transcriptase combined with random primers. The method was performed as previously described [19], [29]. The samples were stored at −20°C or immediately used.

### Whole Transcriptome Amplification

For viral RNA amplification we used the WTA method [29] with the QuantiTect WTA kit (Qiagen), except for the reverse transcription step that was replaced by one of the three RT methods described above. We also modified the protocol by performing amplification at 30°C for 2–8 h. We purified Repli-g amplified DNA according to the supplementary protocol, using the QIAamp DNA Mini Kit (Qiagen), and validated its purity and concentration using a NanoDrop spectrophotometer (Thermo Scientific). The DNA was stored at −80°C or immediately used. To avoid contamination between samples, we adopted precautions normally used during routine viral diagnostic PCR analysis at SSI, where extraction, amplification and analyses are physically separated and negative samples are included in all steps.

### Quantification and Confirmation by Real-time PCR

The technique used for routine diagnostic virus analysis at SSI is quality-assured real-time PCR (ISO 17025; 2005, SSI). To confirm presence of virus in the samples and quantify the virus before and after WTA, we performed virus-specific real-time PCR. We used in-house assays for DENV, WNV, orthopoxvirus, parapoxvirus, Usutu virus, Hantaan virus, Toscana virus, BK virus (BKV) and rotavirus A; and previously published assays for JC virus (JCV) [30], cowpox and monkeypox viruses [31], Chikungunya virus [32], Eastern equine encephalitis virus (EEEV) [33], JEV [34], TBEV [35], YFV [36], Lassa virus and CCHFV [37], Dobrava-Belgrade virus (DOBV) [38], Puumala virus [39], Rift Valley fever virus (RVFV) [36] and Marburg virus [40]. PCR was performed using an Mx3005P (Stratagene) thermal cycler. We calculated the fold difference in concentration from the ΔC_t_ obtained from real-time PCR before and after WTA, combined with dilution factors. Here we made the assumption that 1 cycle change in C_t_-value was equivalent to a doubling of target DNA. We estimated the sample concentrations of the HCV-positive, DENV-positive and WNV-positive clinical samples by performing a series of 10-fold dilutions of the HCV-positive serum sample (1.2×10^6^ IU/ml), the DENV13-01 QCMD sample (1.0×10^6^ copies/ml) and the WNV13-01 QCMD sample (1.0×10^7^ copies/ml), under the assumption that no viral NA was lost during purification.

### Microarray Analysis

We analysed samples with the LLMDAv2 microarray, developed at the Lawrence Livermore National Laboratory (LLNL), USA and described elsewhere [14], [19], [41]. The LLMDAv2 contains 388,000 oligonucleotides probes designed from all sequenced viruses and bacteria [14]. Labelling and microarray hybridization was performed according to manufacturer protocols (Gene expression analysis, Roche NimbleGen) with the exception that 8 µg, instead of 2 µg, of labelled material was used for hybridization. Microarray data was analysed using a simple Excel-based data analysis method developed at SSI (SSI analysis) as described previously [19]. Since the SSI analysis is not optimized for bacteria, any bacterial hits were excluded from the results. Non-human, non-zoonotic pathogens were also excluded since they are assumed to be clinically irrelevant in a diagnostic setting. Additional data analyses were performed on the samples using the CLiMax software developed at LLNL and described elsewhere [14], [41].

Microarray data were submitted to the Gene Expression Omnibus (GEO) database http://ncbi.nlm.nih.gov/geo/with the accession number GSE55576. All microarray data used in this study are MIAME compliant.

## Results

### A Modified WTA Protocol Using 5′-Phosphorylated Random Primers for cDNA Synthesis

**Figure 1:**
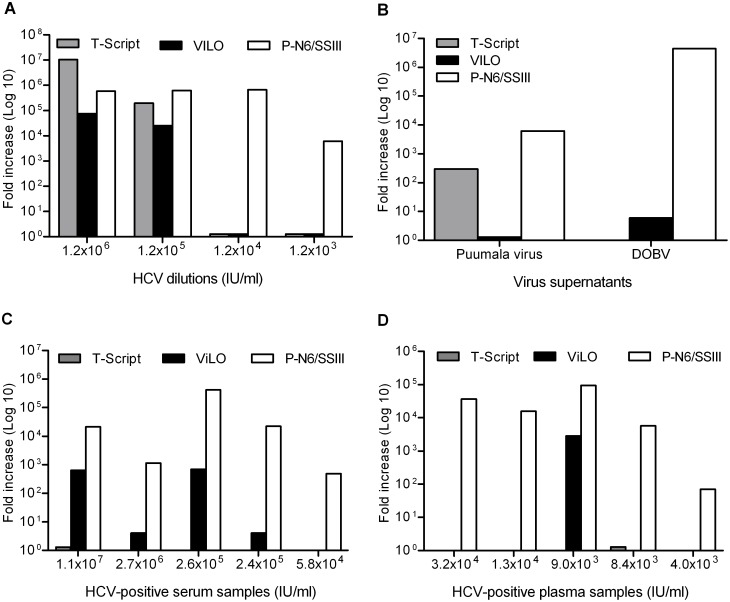
Improved WT amplification when using 5′-phosphorylated random hexamers in RT-reaction. Comparison of three different RT-reactions in the Whole Transcriptome Amplification (WTA) protocol. Purified viral RNA was amplified by WTA using VILO, T-Script or P-N6/SSIII RT-reaction. Virus-specific real-time PCR was performed before and after the amplification step, and fold increase was calculated using ΔC_t_-values and dilution factors for each sample tested. (A) WTA-protocols tested with a 10-fold serial dilution of an HCV-positive serum sample with known concentration. (B) WTA-protocols tested with two different virus-positive cell culture supernatants, Puumala virus and Dobrava-Belgrade virus (DOBV), respectively. (C) WTA-protocols tested with five HCV-positive serum samples with estimated concentration (IU/ml). (D) WTA-protocols tested with five HCV-positive plasma samples with estimated concentration (IU/ml).

To enable successful microarray identification of virus in clinical samples, we have previously used the Phi29 polymerase-based WTA method (Qiagen) [19], [29]. The WTA protocol includes three sequential reactions: a reverse transcription reaction to generate cDNA, ligation of cDNA fragments into large linear chains, and amplification by the Phi29 polymerase [29]. To assure an efficient ligation, we replaced the included RT reaction with Superscript III and 5′-phosphorylated random hexamers (P-N_6_) hereafter called P-N6/SSIII. This was done in order to phosphorylate the 5′-end of the cDNA fragments so that new phosphodiester bonds could be formed during the ligation step [42], [43]. We compared this method to the manufacturers RT reaction (T-Script using random and oligo-dT primers) and to RT using Superscript VILO cDNA kit [19], [29]. Prior to RT and amplification, samples were pre-treated according to a previously described protocol [19]. The different RT protocols were tested in parallel on 10-fold serial dilutions of an HCV-positive serum sample (1.2×10^6^ IU/ml) (Figure 1A), on two supernatants containing the hantaviruses Puumala virus and DOBV, respectively (Figure 1B), and on 10 HCV-positive clinical samples with varying viral concentrations (Figure 1C–1D). For all samples tested, whole transcriptome (WT) amplification of cDNA generated by P-N6/SSIII was more efficient than VILO or T-Script. Therefore, the P-N6/SSIII RT-reaction was used for all further WT amplifications.

### WT Amplification of Emerging Virus in Non-clinical Samples

**Figure 2:**
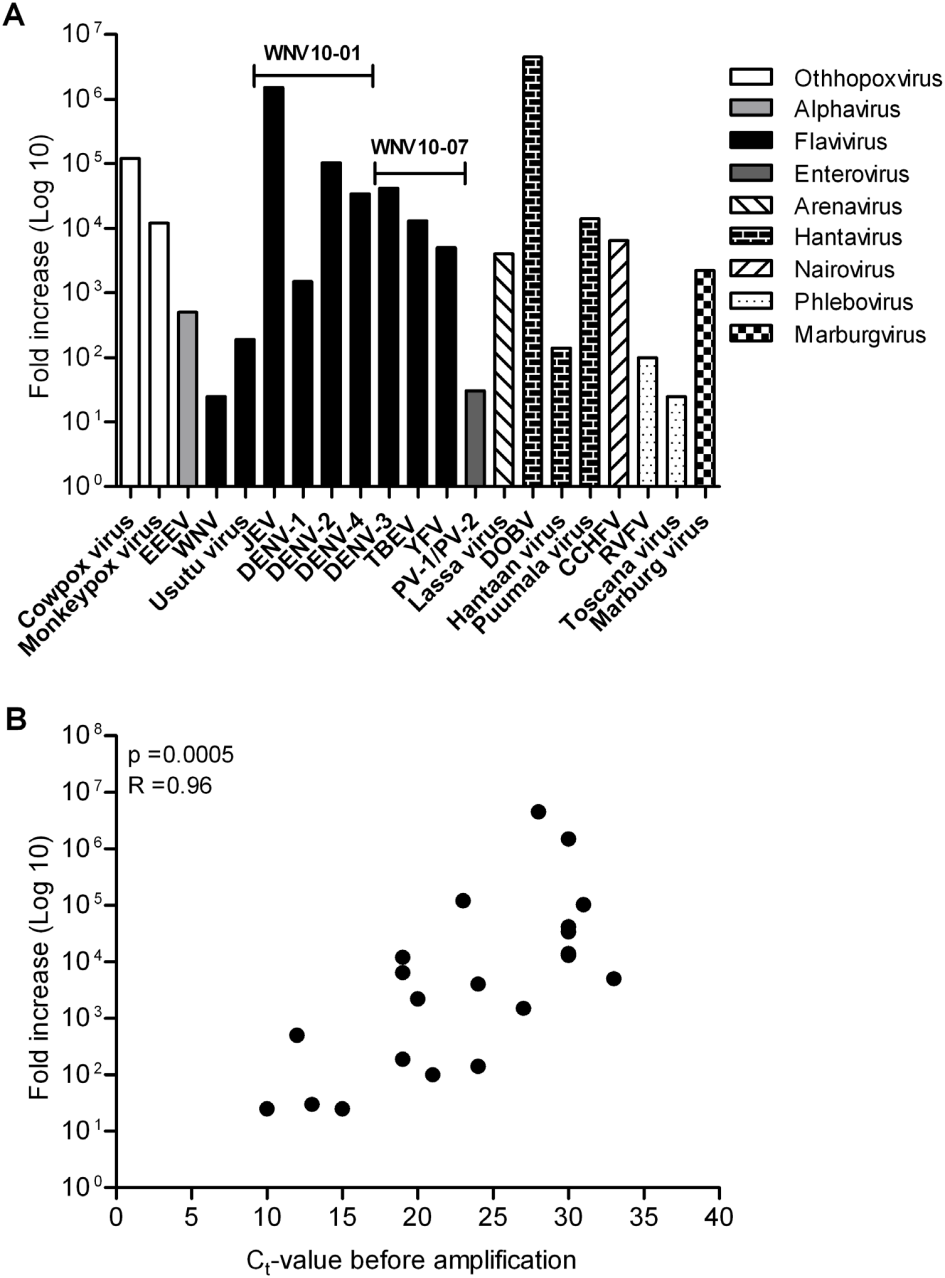
Modified WT amplification of non-clinical samples containing emerging virus. Purified viral RNA from a wide range of virus-positive cell culture supernatants (SN) or QCMD panel samples was amplified by WTA using the P-N6/SSIII RT-reaction. Virus-specific real-time PCR was performed before and after the amplification step, and fold increase was calculated using ΔC_t_-values and dilution factors for each sample tested. (A) Fold increase of WT-amplified emerging viruses belonging to different virus genera. The two QCMD panel samples (WNV10-01 and WNV10-07) containing mixtures of different flaviviruses are highlighted. (B) The correlation between fold increase in WT amplification and viral sample content (C_t_ before WT amplification).

WT amplification using the P-N6/SSIII RT-method was tested for its ability to amplify emerging viruses. Due to difficulty in getting access to clinical samples positive for a diverse set of emerging viruses, we initially tested the method on a wide range of virus-positive cell culture supernatants (SN), purified viral NA or QCMD panel samples (Table S1). The WT amplification was analysed using virus specific real-time PCRs before and after amplification (Table S1 and Figure 2). For all samples tested, amplification of the emerging virus was observed (Figure 2A). For EEEV (Alphavirus), Usutu virus (Flavivirus), WNV (Flavivirus), PV (Enterovirus), Hantaan virus (Hantavirus), RVFV (Phlebovirus) and Toscana virus (Phlebovirus) the amplification was relatively small with a fold increase between 25–500 (Table S1 and Figure 2A). However, for other samples much larger fold increases were observed, such as JEV (Flavivirus) with a fold increase of 1.5×10^6^, DOBV (Hantavirus) with a fold increase of 4.5×10^6^ and Puumala virus (Hantavirus) with a fold increase of 1.4×10^4^ (Table S1 and Figure 2A). When we examined the relationship between amplification (fold increase) and viral content (C_t_-values before WT amplification) (Figure 2B), we observed a significant correlation between WT amplification and viral content. Samples containing a high viral content were amplified to a lesser extent than samples containing a lower viral content, which could reflect that for samples with a high concentration of NA, primers and nucleotides are depleted quickly, resulting in a lower WT amplification.

To investigate whether the presence of several viruses in a sample would interfere with WT amplification, we tested two QCMD panels of samples (WNV10-01 and -07) containing mixtures of four and three different flaviviruses, respectively, each at a concentration of 1.0×10^6^ copies/ml (Table S1 and Figure 2A). All seven flaviviruses were WT amplified; however DENV-1 was amplified to a lower degree than to the other DENV subtypes or flaviviruses (Figure 2A). This most likely reflect a difference in the sensitivity of the Dengue subtype-specific primers used to analyse the WT amplification rather than virus subtype-specific variation in the WT amplification. In summary, the modified WT amplification method was able to amplify emerging viruses in 21 different non-clinical samples.

### Microarray Detection Range

To test the sensitivity of the previously described LLMDA microarray [14], [19], [26] for RNA viruses, we performed microarray analysis on a 10-fold dilution series of a HCV-positive serum sample (1.2×10^6^ IU/ml), a RVFV-positive supernatant (3.3×10^6^ copies/ml), a WNV-positive QCMD panel sample (WNV13-01) (1.2×10^7^ copies/ml), a DENV-positive QCMD panel sample (DENV13-01) (1.0×10^6^ copies/ml) and two QCMD panel samples (WNV13-10 and WNV13-11) containing mixtures of JEV, DENV-1, DENV-2, DENV-4 and YFV, DENV-3, TBEV respectively (1.0×10^6^ copies/ml) (Table 1). Dilutions ranging from 10^6^ to 10^2^ copies/ml were WT amplified, labelled and hybridised to the LLMDAv2 microarray. Microarray analysis was performed using the SSI [19] and CLiMax data analysis methods [14], [41] (data not shown).

For the HCV, RVFV and WNV samples, dilutions of 10^6^ to 10^3^ copies/ml yielded sufficient viral material for successful identification by the LLMDAv2, while dilutions of 10^2^ copies/ml were not detected by the microarray (Table 1). Dilutions of 10^2^ copies/ml will theoretically result in an input of 24 copies to the RT-reaction and 12 copies to the WTA-reaction. Analyses of the viral concentrations after WT amplification of the non-detectable 10^2^ copies/ml dilutions showed that RVFV, HCV and WNV were amplified to 1.7×10^5^, 4.3×10^5^ and 4.4×10^5^ copies/ml, respectively (Table 1).

The detection limit for DENV, JEV and TBEV was 10^4^ copies/ml (Table 1) and the detection limit for YFV was higher (10^5^ copies/ml) than the rest of the flaviviruses analysed. The WNV13-11 sample was documented as containing YFV, DENV-3 and TBEV, each at 1.0×10^6^ copies/ml; however, analysis of the C_t_-values of YFV and TBEV before amplification showed a higher value for YFV (C_t_ = 27) compared to TBEV (C_t_ = 23) (Table 1), which could indicate a lower viral content of YFV in the WNV13-11 sample than was documented. Analysis of the viral concentration after WT amplification of the non-detectable 10^3^ copies/ml dilutions showed that DENV, JEV and TBEV were amplified to 3.1×10^5^, 1.0×10^4^ and 2.1×10^4^ copies/ml, respectively (Table 1). From this we conclude that at least 10^3^ copies/ml is needed for a successful amplification with the modified WTA method and at least 10^5^ copies/ml is needed after WT amplification in order to reliably identify viruses with the LLMDAv2. This concentration is equivalent to 0.17 femtomolar, demonstrating exquisite sensitivity of the LLMDAv2 platform.

### Microarray Detection of Emerging Virus in Non-clinical Samples

The LLMDA microarray [14], [19], [26] was tested for its ability to correctly identify a wide range of virus-positive cell culture supernatants (SN), purified viral NA or QCMD panel samples containing emerging viruses (Table 2). The WT amplified samples previously described (Table S1) were labelled and hybridised to the LLMDAv2 microarray. Microarray analysis was performed using the SSI [19] and CLiMax data analysis methods [14], [41].

In all 21 samples analysed, both methods identified the correct virus (Table 2). In more than half of the samples, human endogenous retroviruses (HERV) were also found (Table 2), consistent with the presence of human host DNA. The CLiMax method identified additional retroviruses such as baboon endogenous virus (BEV), simian retrovirus 1 (SRV-1), *Mus dunni* endogeneous virus (MDEV), murine leukemia virus (MuLV) and mouse mammary tumour virus (MMTV). These additional viruses were not identified by the SSI method because non-human, non-zoonotic pathogens were considered clinically irrelevant and excluded in the SSI data analysis.

For four of the samples (cowpox virus, monkeypox virus, PV-1/PV-2 and Usutu virus), the SSI method had difficulties in distinguishing between different genus-members and subtypes. In the Usutu virus sample (Flavivirus), the SSI method identified both Usutu virus and JEV, another Flavivirus species, as being present, while the CliMax analysis correctly identified Usutu virus only. In the PV sample, the CLiMax analysis correctly identified PV-1 and PV-2, while the SSI analysis made an additional false-positive detection of PV subtype 3 (Table 2). In the samples of cowpox virus and monkeypox virus, both methods identified additional members of the *Orthopoxviridae* family as present. The SSI analysis identified both samples as mixtures of cowpox, monkeypox, vaccinia and variola viruses, while the CliMax analysis identified the correct cowpox or monkeypox virus together with the variola minor virus (Table 2), which belongs to the same genus. Detailed examination of the probes with positive signals (greater than the 99^th^ percentile of the negative control intensities) showed that all such probes with alignments to the variola minor virus genome had strong matches in the cowpox and monkeypox genomes; so that the identification of variola minor virus in the CliMax analysis in these samples is most likely due to cross-hybridization of these probes.

**Figure 3:**
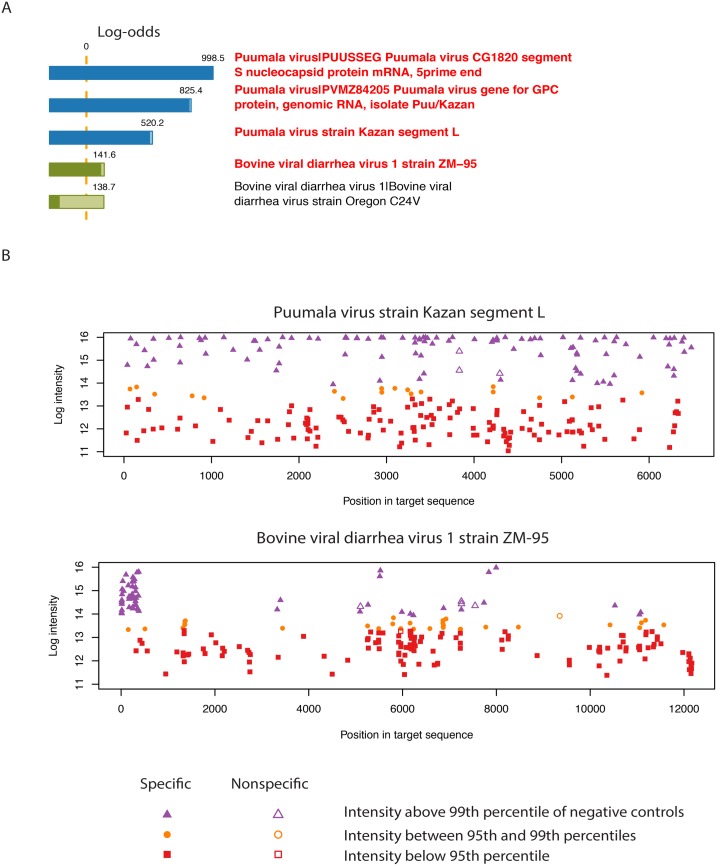
CLiMax analysis detects Puumala virus in a non-clinical sample. The results of microarray analyses of WT-amplified viral DNA-samples, using CLiMax analysis. (A) Log-odds scores for a Puumala virus-positive sample. The lighter and darker-coloured portions of the bars represent the unconditional and conditional log-odds scores, respectively. The conditional log-odds scores shows the contribution from a target that cannot be explained by another, more likely target above it, while the unconditional score illustrates that some very similar targets share a number of probes. (B) Target sequence-probe alignment plots for segment L of the Puumala virus genome and for BVDV-1, showing probe intensity vs probe position in the viral genome. Plot symbol and color indicates positive (>99^th^ percentile), negative (<95^th^ percentile), or equivocal hybridisation signals; hollow symbols indicate probes found to hybridise non-specifically. The pattern seen for BVDV-1, in which positive probes are restricted to a few narrow genome regions, is a typical cross-hybridisation result.

For five samples (Hantaan virus, Puumala virus, RVFV, Naples virus and Marburg virus) the CLiMax analysis identified additional viruses that were not observed using the SSI analysis (Table 2). To better understand the source of these additional predictions, we used the CliMax software to generate sequence-probe alignment plots, where the intensity of each probe is plotted against its alignment position in the viral genome. These plots clarify whether identification of a virus is based on presence of the whole genome or may be due to cross-hybridization from probes matching sub-regions of other genomes present in the sample. For example, the sequence-probe alignment plots for the Puumala virus sample show the positive probes to be uniformly distributed across all three Puumala virus genome segments, indicating the presence of the whole viral genome (Figure 3B, top). Probe hits for the bovine viral diarrhea virus 1 (BVDV-1) genome show a different pattern, landing in only a narrow region suggesting nonspecific- or cross-hybridisation (Figure 3B, bottom). We refer to this pattern as a fragmented alignment plot.

We observed a similar fragmented alignment pattern for RVFV segment S in the Marburg sample, indicating non-specific cross-hybridisation (data not shown). In contrast, we obtained uniform positive probe distributions for mammalian orthoreovirus 1 and 3 (MRV) genomes in the Hantaan virus sample, for CCHFV and Simian virus 5 (SV5) genomes in the RVFV sample, and for BVDV-1 in the Naples virus sample, indicating that these complete viral genomes were truly present (data not shown). CCHFV specific PCR could not confirm the presence of CCHFV in the RVFV sample (data not shown). The other additional findings were all considered clinically irrelevant and therefore not further investigated by PCR.

The presence of several viruses in a sample did not interfere with identification, as can be seen by the microarray analysis of the two panels of samples (WNV10-01 and -07) containing mixtures of different flaviviruses (Table 2). Microarray analysis correctly identified all four viruses present in WNV10-01 and all three viruses present in WNV10-07 (Table 2). However, the individual DENV subtypes were difficult to distinguish completely. In the WNV10-10 sample the CLiMax analysis identified DENV type 3, and in the WNV10-07 sample both analysis methods detected DENV type 1 and 2. These extra DENV findings were later confirmed as false-positives by Dengue subtype-specific PCR (data not shown). In addition, the SSI analysis of the WNV10-07 sample identified Omsk haemorrhagic fever virus (OHFV), which also belongs to the Flavivirus genus [44]. This finding was not observed using the CLiMax analysis and hence not checked by PCR. The CLiMax analysis also found HERV, rotavirus A and porcine rotavirus C in both samples as well as BVDV-1 in WNV10-01 and SV5 in WNV10-07. The presence of rotavirus A was confirmed by rotavirus A-specific PCR (data not shown). BVDV, SV5 and porcine rotavirus C were considered clinically irrelevant and therefore not confirmed by PCR.

In summary, the LLMDAv2 correctly identified single and multiple viruses present in non-clinical samples with a very low level of false positive signals. The CLiMax analysis method identified every virus present in the samples whereas the simpler SSI analysis method only identified clinically relevant human pathogens.

### Microarray Detection of Emerging Viruses in Clinical Samples

**Figure 4:**
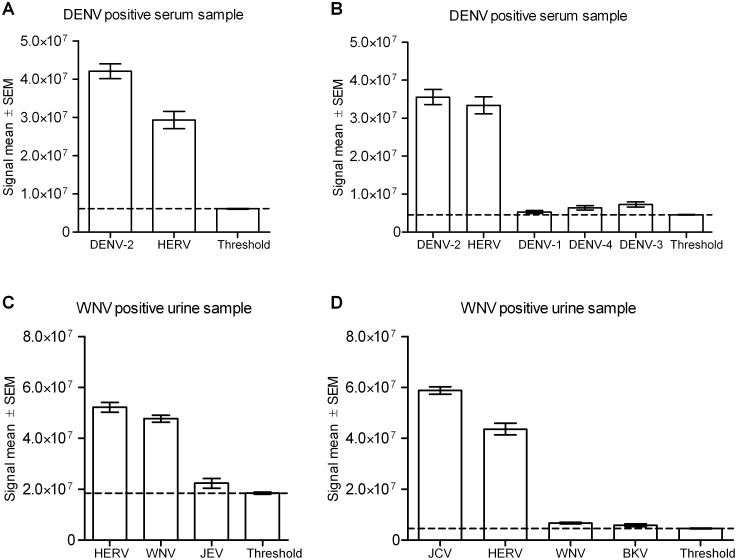
Microarray analysis correctly identifies emerging viruses in clinical samples. The results of microarray analysis of WT-amplified virus-positive clinical samples, using the SSI analysis method. Graphs show the signal mean for the probe intensities for each detected virus. The bar across the graph demonstrates the signal threshold at the 99^th^ percentile of the random control intensities. (A) Microarray analysis of a Dengue-positive serum sample. (B) Microarray analysis of another Dengue-positive serum sample. (C) Microarray analysis of a WNV-positive urine sample. (D) Microarray analysis of another WNV-positive urine sample.

We tested the LLMDAv2 microarray on 18 clinical samples previously identified as positive by real-time PCR for emerging viruses. The correct virus was identified in 17 samples using both the SSI (Table 3) and CLiMax analyses (data not shown). The sample identified only as a parapoxvirus was determined to be Orf virus, a member of the Parapoxvirus genus. Seven of the eight DENV-positive samples were clearly determined by the microarray analysis to be positive for DENV type 2, DENV type 1 or DENV type 3. DENV type 4 was not identified in any of the clinical samples. Additional DENV subtypes were detected in four of the samples, but at very low probe signal intensities compared to the correct DENV subtype probe signal (Figure 4A+4B). These were confirmed as negative by Dengue subtype-specific PCR (data not shown). One DENV-positive sample was also positive for hepatitis GB virus C (GBV-C). One DENV-positive sample was not identified by the microarray. Six urine samples were positive for WNV and two of these samples were identified as having additional viruses (Table 3). One WNV sample was also positive for the polyomaviruses JCV and BKV (Figure 4C), which later were confirmed as present by PCR (data not shown). Another WNV sample was positive for JEV (Figure 4D), but this finding could not be confirmed by PCR (data not shown). In addition, the microarray detected HERV in almost all samples, consistent with the presence of human DNA, and the common Torque Teno virus (TTV) [19], [23], [45] in the CCHFV and two DENV samples. Virus-negative urine, CSF and serum were also analysed and confirmed to be negative for virus (Table 3), except for HERV found in the CSF sample. In summary, the LLMDAv2 correctly identified emerging viruses present in 17 of the 18 clinical samples analysed. The only sample not identified was a DENV-positive sample, in which the viral concentration was determined to be below the detection limit, as described below.

**Table 3:** Microarray results on clinical samples containing emerging viruses.

Group*	Genus	Virus	Sample	Detected virus (SSI analysis)	ΔC_t_ a	Fold increase^b^	After WTA^c^	Sample conc^c^
dsDNA	Parapoxvirus	Parapox sp.	skin lesion	**Orf** 95	45–23	3.6×10^6^	ND	ND
(+) ssRNA	Alphavirus	Chikungunya	Serum	**Chikungunya virus**	30–24	3.5×10^4^	ND	ND
	Flavivirus	DENV	Serum	**DENV-2,** DENV-4, DENV-3, DENV-1, HERV	23-ND	-	ND	ND
		DENV	Serum	**DENV-2,** HERV, TTV	29-ND	-	ND	ND
		DENV	Serum	**DENV-1, GBV-C,** DENV-3, DENV-4, DENV-2, JEV, HERV, TTV	24–8	1.5×10^6^	2.5×10^12^	1.8×10^7^
		DENV	Serum	**DENV-3,** DENV-1, DENV-4, DENV-2, HERV	25–12	1.7×10^5^	2.7×10^11^	1.1×10^7^
		DENV	Serum	**DENV-1**	26–13	2.4×10^5^	1.6×10^11^	5.1×10^6^
		DENV	serum	**DENV-1**	31–16	8.5×10^5^	2.8×10^10^	3.2×10^5^
		DENV	serum	**DENV-1,** DENV-3, DENV-4, DENV2, HERV	34–23	3.2×10^4^	5.3×10^8^	8.3×10^4^
		DENV	serum	HERV	37–38	15	1.4×10^5^	1.1×10^4^
		WNV	urine	**WNV**, HERV	28–26	133	2.8×10^7^	4.9×10^5^
		WNV	urine	**WNV**, HERV, JCV, BKV	30–28	73	8.6×10^6^	2.2×10^5^
		WNV	urine	**WNV,** HERV	31–29	103	6.4×10^6^	1.3×10^5^
		WNV	urine	**WNV,** HERV	31–31	32	2.9×10^6^	1.3×10^5^
		WNV	urine	**WNV,** HERV	32–36	1	3.0×10^5^	8.9×10^4^
		WNV	urine	**WNV,** JEV, HERV	34–26	7.4×10^3^	2.5×10^7^	3.7×10^4^
(−) ssRNA	Nairovirus	CCHFV	serum	**CCHFV**, HERV, TTV	ND	-	ND	ND
	Phlebovirus	Toscana	CSF	**Toscana virus**, HERV	31	25	-	2.4×10^3^
-	-	Neg. ctrl	Urine	-	ND	-	-	-
-	-	Neg. ctrl	CSF	HERV	ND	-	-	-
-	-	Neg. ctrll	serum	-	ND	-	-	-

**NOTE**. Conc, Concentration; DENV; Dengue virus, WNV, West Nile virus; JCV, JC polyomavirus; BKV, BK polyomavirus; CCHFV, Crimean-Congo haemorrhagic fever virus; Neg. ctrl, Negative control; GBV-C, hepatitis GB virus C; JEV, Japanese encephalitis virus; CSF, Cerebrospinal fluid; HERV, human endogenous retrovirus; TTV, torque teno virus; ND, not determined. Bold represents correctly identified virus.

*Viruses are grouped based on nucleic acid content, according to the Baltimore Classification.

95 results obtained using a 95 percentile threshold.

a Difference in C_t_-value in real-time PCR before and after WT amplification.

b Fold increase after WT amplification, calculated from ΔC_t_ combined with dilution factors for each sample.

c Copies/ml (HCV; IU/ml).

To assess viral concentration in clinical samples, we performed specific real-time PCR before and after WT amplification. We estimated the viral concentration of 6 WNV-positive urine samples and 6 DENV-positive serum samples by comparison to PCR results for the series of 10-fold dilutions of the QCMD panel WNV and DENV samples (Table 2 and Table 3). The WNV-positive urine samples were determined to have concentrations between 3.7×10^4^ and 4.9×10^5^ copies/ml before WTA and concentrations between 3.0×10^5^ and 2.8×10^7^ copies/ml after WT amplification (Table 3). These samples all had concentrations above the detection limit (10^3^ copies/ml) determined for dilutions of the WNV-positive QCMD sample (WNV13-01) (Table 1). Analysis of the WT amplification showed that WNV from urine samples was not amplified as efficiently as WNV from the QCMD sample (Table 1 and Table 3), however the concentration after WTA was still above 10^5^ copies/ml and hence detectable by the LLMDAv2.

The DENV-positive serum samples were determined to have concentrations between 1.1×10^4^ and 1.8×10^7^ copies/ml before WT amplification and between 1.4×10^5^ and 2.5×10^12^ copies/ml after WTA (Table 3). The DENV-positive sample which was not detected by the microarray had an estimated concentration of 1.1×10^4^ copies/ml, which was near the pre-amplification detection limit seen for dilutions of the QCMD DENV sample (10^4^ copies/ml) (Table 1 and Table 3); and a concentration after WTA of 1.4×10^5^ copies/ml, which is near the post-WTA limit of detection (10^5^ copies/ml). This sample was also near the limit of detection with real-time PCR, with a C_t_ value of 37 before amplification. In summary, 11 out of 12 clinical samples analysed had viral concentrations above the detection limit of the LLMDAv2.

## Discussion

The disease symptoms for emerging viruses are often similar to those of other more common viruses, posing a diagnostic challenge to clinicians unfamiliar with the novel organism. In the case of emerging viruses it is crucial for patient treatment and for containment of a potential epidemic to quickly identify the correct virus. We demonstrate the ability of the LLMDAv2 array combined with a modified WTA protocol to correctly identify 29 different emerging viruses in both clinical and non-clinical samples. Previously we have also shown that LLMDAv2 can detect a broad range of common viruses in clinical samples [19]. We show a sensitivity of 10^3^–10^4^ copies/ml for different emerging RNA viruses, which is in the range of clinical relevance, but not as sensitive as specific real-time PCR. However, the use of PCR requires a specific hypothesis as to the causative agent, which is not the case with the LLMDA array. We use a modified random WTA method to amplify the RNA virus and show that least 10^5^ copies/ml of amplified material is needed in order to have a successful identification by the LLMDAv2. This is equivalent to the recently published data that show detection of 10^5^ copies of vaccinia virus DNA without any amplification prior to hybridization to the 4x72K version of the LLMDA [46].

The samples used in this study to measure sensitivity were all dilutions of viral samples or supernatants and do not represent clinical samples containing low viral concentrations. Therefore, further experiments to investigate clinical sensitivity are warranted. Previous reports have shown high clinical sensitivity (86–97%) and specificity (98–99%) of another microarray, the Virochip [15], when it was applied to samples from different respiratory virus infections that were confirmed by specific PCR [20], [21]. In our study, we correctly identified emerging viruses in 17 out of 18 clinical samples that were positive by specific PCR, corresponding to a clinical sensitivity of 94%. However, this study must be considered preliminary due to its small size. We are currently comparing the LLMDAv2 against standard diagnostic real-time PCR tests for a wide range of viruses and clinical sample materials. However, our ability to compare diagnostic assays for emerging viruses is limited due to the relatively small number of clinical samples received at SSI containing these viruses.

Overall, the LLMDAv2 demonstrates high specificity and sensitivity with few false positives. The majority of additional hits found by the microarray data analysis are retroviruses normally found in mammalian genomes (HERV, BEV, MDEV, MuLV and MMTV). They are clinically irrelevant and most probably originate from host or cell culture DNA. The BEV identified in the Ebola virus, cowpox virus and monkeypox virus SN samples is not surprising, since cross-hybridization of endogenous retroviruses in African green monkey-derived Vero E6 cell cultures to the BEV probes has been previously reported [26]. The MDEV, MuLV and MMTV identified in the poliovirus sample are consistent with the fact that PV is cultured in mouse-derived L20B cells. In a few samples (Usutu virus, cowpox virus, monkeypox virus, RVFV, Marburg virus, the WNV10-panel samples, one clinical DENV sample, and one clinical WNV sample), additional viruses were identified that predominantly belonged to the same family or genus as the correct virus. All of them were determined to be false positives by virus-specific PCR indicating a need to improve the specificity of the probes or the analysis methods. Both data analysis methods had difficulty in distinguishing between the four different DENV subtypes (Table 1 and Figure 3B). This was not surprising, since viral strain subtyping was not a goal of the LLMDAv2 design [14]. Nevertheless, our work shows that improvements to LLMDA probe specificity are needed to increase its value for diagnosis and outbreak detection.

The CLiMax software is numerically intensive and requires a large-memory LINUX server harbouring a library of probe-target binding probabilities that are the basis for pathogen identification [14], [41]. The CLiMax analysis is sophisticated and powerful, providing a user-friendly web interface to a database that keeps track of requested analyses and their results. In addition to a list of probable viruses, the CLiMax software can generate a target sequence-probe alignment plot showing probe fluorescence intensities together with the location of probe hits across each viral genome detected. This can help to distinguish the presence of whole viral genomes from non-specific probe hits and cross-reactivity.

The analysis developed in-house at SSI processes microarray feature intensities produced by the NimbleScan software within a Microsoft Excel framework [19]. While the CLiMax analysis is designed for broad-spectrum detection of all microbial targets represented on the LLMDA, the Excel-based SSI analysis is more focused toward identification of human-infecting viral pathogens. The relative simplicity of the SSI analysis is attractive for a clinical diagnostic environment, since it requires less costly computing hardware, and provides a clearer diagnostic result for clinicians, because clinically irrelevant non-human and non-zoonotic pathogens are excluded from the analysis. The CLiMax software is a more sophisticated, precise tool for data analysis in a research environment. Its ability to identify microbial pathogens from all host species makes this analysis method ideal for analysis of special cases such as detection of novel zoonotic viruses and research purposes.

## Supporting Information

Table S1 **Modified WT amplification of non-clinical samples.**
(DOCX)
