# Supplementary material for: The Microbial Detection Array for Detection of Emerging Viruses in Clinical Samples - A Useful Panmicrobial Diagnostic Tool
Source: PLoS One. 2014 Jun 25;9(6):e100813. doi: 10.1371/journal.pone.0100813 (PMC4070998; doi:10.1371/journal.pone.0100813)
Supplement: Table S1 — Modified WT amplification of non-clinical samples. (DOCX) [file pone.0100813.s001.docx]

**Table S1**. **WT amplification of non-clinical samples**

| **Group^*^** | **Genus** | **Sample** | **Virus** | **∆C_t_^+^** | **Fold incr.^‡^** |
| --- | --- | --- | --- | --- | --- |
| dsDNA | Orthopoxvirus | pur. DNA | Cowpox | 23-11 | 1.2x10^5^ |
|  |  | Pur. DNA | Monkeypox | 19-11 | 1.2x10^4^ |
| (+)ssRNA | Alphavirus | SN | EEEV | 12-7 | 500 |
|  | Flavivirus | pur. RNA | Usutu | 15-12 | 190 |
|  |  | pur. RNA | WNV | 19-19 | 25 |
|  |  | WNV10- 01 | JEV | 30-14 | 1.5 x 10^6^ |
|  |  |  | DENV-2 | 31-19 | 1.0x10^5^ |
|  |  |  | DENV-1 | 27-21 | 1.5x10^3^ |
|  |  |  | DENV-4 | 30-20 | 3.4x10^4^ |
|  |  | WNV10- 07 | TBEV | 30-21 | 1.3x10^4^ |
|  |  |  | DENV-3 | 30-19 | 4.2x10^4^ |
|  |  |  | YFV | 33-25 | 5.0x10^3^ |
|  | Enterovirus | SN | PV-1, PV-2 | 13-12 | 30 |
| (-)ssRNA | Arenavirus | SN | Lassa | 24-16 | 4.0x10^3^ |
|  | Hantavirus | SN | DOBV | 28-11 | 4.5 x 10^6^ |
|  |  | SN | Hantaan | 24-21 | 140 |
|  |  | SN | Puumala | 30-21 | 1.4x10^4^ |
|  |  | SN | Seoul | ND | - |
|  |  | SN | Sin Nombre | ND | - |
|  | Nairovirus | SN | CCHFV | 19-11 | 6.4x10^3^ |
|  | Phlebovirus | SN | RVFV | 21-19 | 100 |
|  |  | SN | Naples | ND | - |
|  |  | SN | Sicilian | ND | - |
|  |  | SN | Toscana | 10-10 | 25 |
|  | Ebolavirus | SN | Ebola Zaire | ND | - |
|  | Marburgvirus | SN | Marburg | 20-14 | 2.2x10^3^ |

**NOTE**. EEEV, eastern equine encephalitis virus; WNV, West Nile virus; CCHFV, Crimean-Congo haemorrhagic fever virus; RVFV, Rift-Valley fever virus; TBEV, Tick borne encephalitis virus; YFV, yellow fever virus; PV, poliovirus; JEV, Japanese encephalitis virus; DENV, Dengue virus; pur. DNA, purified DNA; SN, cell culture supernatant; pur. RNA, purified RNA; WNV10-01, sample from QCMD EQA WNV panel 10-01; WNV10-07, sample from QCMD EQA WNV panel 10-07

*Viruses are grouped based on nucleic acid content, according to the Baltimore Classification

+Difference in C_t_-value in real-time PCR before and after WT amplification

‡Fold increase after WT amplification, calculated from ∆C_t_ combined with dilution factors for each sample
